# Serum Metabolites as an Indicator of Developing Gestational Diabetes Mellitus Later in the Pregnancy: A Prospective Cohort of a Chinese Population

**DOI:** 10.1155/2021/8885954

**Published:** 2021-02-05

**Authors:** Mengyuan Tian, Shujuan Ma, Yiping You, Sisi Long, Jiayue Zhang, Chuhao Guo, Xiaolei Wang, Hongzhuan Tan

**Affiliations:** ^1^Xiangya School of Public Health, Central South University, Changsha, China; ^2^Hunan Key Laboratory of Clinical Epidemiology, Changsha, China; ^3^Clinical Research Center for Reproduction and Genetics in Hunan Province, Reproductive and Genetic Hospital of CITIC-Xiangya, Changsha, China; ^4^Department of Obstetrics, Hunan Provincial Maternal and Child Health Hospital, Changsha, China

## Abstract

**Objective:**

Gestational diabetes mellitus (GDM) is a common metabolic disorder with onset during pregnancy. However, the etiology and pathogenesis of GDM have not been fully elucidated. In this study, we used a metabolomics approach to investigate the relationship between maternal serum metabolites and GDM in early pregnancy.

**Methods:**

A nested case-control study was performed. To establish an early pregnancy cohort, pregnant women in early pregnancy (10‐13^+6^ weeks) were recruited. In total, 51 patients with GDM and 51 healthy controls were included. Serum samples were analyzed using an untargeted high-performance liquid chromatography mass spectrometry metabolomics approach. The relationships between metabolites and GDM were analyzed by an orthogonal partial least-squares discriminant analysis. Differential metabolites were evaluated using a KEGG pathway analysis.

**Results:**

A total of 44 differential metabolites were identified between GDM cases and healthy controls during early pregnancy. Of these, 26 significant metabolites were obtained in early pregnancy after false discovery rate (FDR < 0.1) correction. In the GDM group, the levels of L-pyroglutamic acid, L-glutamic acid, phenylacetic acid, pantothenic acid, and xanthine were significantly higher and the levels of 1,5-anhydro-D-glucitol, calcitriol, and 4-oxoproline were significantly lower than those in the control group. These metabolites were involved in multiple metabolic pathways, including those for amino acid, carbohydrate, lipid, energy, nucleotide, cofactor, and vitamin metabolism.

**Conclusions:**

We identified significant differentially expressed metabolites associated with the risk of GDM, providing insight into the mechanisms underlying GDM in early pregnancy and candidate predictive markers.

## 1. Introduction

Gestational diabetes mellitus (GDM), a common metabolic disorder during pregnancy, is defined as glucose intolerance occurring in the second and third trimesters, resulting in varying degrees of hyperglycemia [1]. Owing to the increase in prevalence, negative economic impacts, and adverse health outcomes of GDM, research focused on GDM has increased. The exact prevalence of GDM worldwide remains unclear [1]; however, in the context of the increasing global prevalence of obesity and diabetes, the prevalence of GDM has increased annually [2], particularly in China [3]. For example, based on a survey of 105473 pregnant women in Tianjin, the prevalence of GDM increased threefold from 2.4% to 6.8% between 1999 and 2008 [4]. GDM has short- and long-term adverse health effects on women and their offspring. During pregnancy, GDM can increase the probability of obstetric complications, such as gestational hypertension, postpartum hemorrhage, dystocia, and abortion, and can lead to a higher incidence of macrosomia, preterm birth, and fetal malformation [5–7]. Furthermore, women who have had GDM have an increased risk of type 2 diabetes mellitus (T2DM), metabolic syndrome, fatty liver, and cardiovascular disease [7–9]. Furthermore, the risk of impaired glucose tolerance, diabetes, hypertension, obesity, and coronary heart disease in the offspring of women with a history of GDM may be significantly elevated [1, 10, 11].

Epidemiological evidence has shown that a family history of diabetes, prepregnancy obesity, excessive weight gain during pregnancy, and advanced age are the main determinants of GDM [12–15]. Researchers have also focused on the studies on the pathogenesis of GDM. There was evidence that some metabolic disorders, such as *β*-cell dysfunction and insulin resistance, are critical components of the pathophysiology of GDM [16]. In addition, genetic factors, inflammation, adipocytokines, and oxidative stress are also closely related to the pathology of GDM [16]. However, the pathogenesis of GDM has not been fully elucidated, to some extent, which limits the further development of effective prevention strategies and treatment measures for GDM. In recent years, advances in metabolomics technology have provided us with help to explore the pathophysiological mechanisms of underlying metabolic abnormalities of GDM. Briefly, analyses of changes in low-molecular-weight metabolites after exposure to external stimuli enable the identification of novel biomarkers for diseases and improve our understanding of pathogenic mechanisms [17]. More and more researchers have explored the changes of GDM-related metabolites and identified novel biomarkers of GDM from biological specimens. For example, based on a nested case-control study, de Seymour et al. analyzed 48 maternal serum samples using gas chromatography coupled to mass spectrometry (GC-MS) at 20 weeks gestation and found that the serum itaconic acid level is significantly higher in pregnant women with GDM than in healthy pregnant women [18]. Sachse et al. analyzed maternal urine samples from a prospective, multiethnic cohort study using proton nuclear magnetic resonance (^1^H-NMR) spectroscopy and found that the steady increase of urinary lactose concentration is the most significant change in the development of GDM [19]. Liu et al. used an advanced metabolomics platform based on ultraperformance liquid chromatography quadrupole time-of-flight mass spectrometry (UPLC/Q-TOF-MS) and found differences in serum arginine, glycine, and 3-hydroxy-isovalerate carnitine levels between pregnant women with GDM and healthy pregnant women in early pregnancy [20]. However, the results of these metabolomics studies of GDM are inconsistent, which may be due to the differences in the GDM diagnostic criteria used, differences in the use of instrumental methods in metabolomics, differences in the various biological specimens, and the differences in the study population characteristics [21].

In general, there have been few longitudinal metabolomics studies in early pregnancy in the Chinese population, while most of the studies are designed with a case-control study conducted in the second trimester. In fact, longitudinal metabolomics studies are a more powerful approach in identifying metabolite changes and their association with related disease [21]. Based on a longitudinal cohort, Law et al. investigated maternal plasma metabolite changes in early pregnancy in GDM women and found that the levels of a number of polyunsaturated or chemically modified phospholipids in the plasma of pregnant women with GDM were significantly lower than those in healthy controls [22]. Meanwhile, they used the same metabolomics approach to explore the differences in the urinary metabolome of GDM cases and healthy controls and found that hypoxanthine, xanthine, xanthosine, and 1-methylgypoxanthine are all elevated in the urine metabolome of pregnant women with GDM [23]. Zhao et al. performed an untargeted longitudinal metabolomics study and revealed that amino acid metabolism, lipid metabolism, and other pathways might be disrupted prior to the onset of GDM [24]. Early pregnancy is a critical period for the onset of GDM, and metabolite detection during this period is of clinical significance for prognosis prediction and early diagnosis. Previous studies have shown that the most significant metabolite changes between GDM and the control group occurred in the first and/or third trimester of pregnancy, with less significant metabolite changes in the second trimester [21]. In addition, there may be significant differences in metabolomic characteristics between ethnic groups, which may be due to differences in genetics, diet, culture, or gut microbes. Thus, more longitudinal metabolomics studies of GDM in the Chinese population in the first trimester of pregnancy are needed.

In this cohort study with follow-up, we investigated the relationship between early pregnancy maternal serum metabolites and the risk of GDM in a Chinese population using an untargeted HPLC-MS metabolomics approach. We attempted to advance the observation starting point to the onset of GDM and explore the possible metabolic abnormalities in the early stage of GDM, so as to develop effective prevention strategies and treatment measures for GDM in the early stage. Our results identify candidate biomarkers for GDM and associated metabolic pathways.

## 2. Methods

### 2.1. Study Subjects

This nested case-control study was based on an early pregnancy follow-up cohort. The prospective cohort (ChiCTR1900020652) included Chinese women recruited during early pregnancy (10‐13^+6^ weeks) from the Hunan Provincial Maternal and Child Health Care Hospital in Changsha between 2016 and 2017. The inclusion criteria are as follows: (1) single birth; (2) conceived naturally; (3) no history of diabetes, hypertension, thyroid disease, and cardiovascular and cerebrovascular diseases before pregnancy; and (4) no acute infection in the last 2 weeks, and no antibiotics were used during pregnancy. A total of 872 subjects were included in the follow-up cohort. The diagnosis of GDM was based on the IADPSG standard updated by the American Diabetes Association in 2011 [25], with a 75 g routine oral glucose tolerance test at 24–28 weeks of gestation after overnight fasting. Patients with blood glucose levels exceeding 5.1, 10.0, and 8.5 mmol/L, respectively, in fasting plasma glucose, 1 h plasma glucose, and 2 h plasma glucose were diagnosed with GDM. According to the diagnostic criteria, 51 cases of GDM were obtained. The controls were matched (1 : 1) to cases with respect to age (±3 years) and timing of blood collection (±1 week). Finally, 51 patients with GDM and 51 healthy participants were included in the study. The study was approved by the Medical Ethics Committee of Hunan Maternal and Child Health Hospital, China (number: EC201624). All methods were performed in accordance with relevant guidelines and regulations, and all participants provided written informed consent.

### 2.2. Serum Collection and Metabolite Extraction

Blood samples (3–5 mL) were collected from all subjects in the first trimester of pregnancy with the assistance of nurses in the blood collection room of the hospital. The samples were thawed and vortexed for 30 s. For preparation of tissue homogenate, tissue was weighed, gradually added to H_2_O, and homogenized. Cells were added to water and sonicated for 10 min at 4°C. For metabolites, sample volumes of 200 *μ*L were extracted with MeOH : ACN (1 : 1, *v*/*v*). The samples then vortexed for 30 s and sonicated for 10 min. To precipitate proteins, the samples were incubated with MeOH : ACN (1 : 1, *v*/*v*) for 1 h at -20°C, followed by 15 min centrifugation at 20,000 g and 4°C. The resulting supernatant was removed and evaporated to dryness in a vacuum concentrator. The dry extracts were then reconstituted in 40 *μ*L/mg·pro of ACN : H_2_O (1 : 1, *v*/*v*), vortexed for 30 s, and sonicated for 10 min. The extracts were centrifuged for 15 min at 20,000 rpm and 4°C to remove insoluble debris. The supernatants were transferred to HPLC vials and stored at -80°C prior to LC/MS analysis. The quality control (QC) samples were prepared by pooling 10 *μ*L of each sample. The extraction procedure for QC samples was the same as that used for metabolite extraction.

### 2.3. HPLC-MS Analysis

The metabolomics analyses were performed on an Agilent 1260 Infinity HPLC platform (Agilent Technologies Co. Ltd., Palo Alto, California, USA) coupled with a Thermo Scientific Q Exactive Mass Spectrometer (Thermo Fisher Scientific, Waltham, Massachusetts, USA). The data was acquired with DDA (DDA method parameters: full scan range: 60 to 900 (m/z); resolution for MS1 and ddMS2: 70,000 and 17,500, respectively; maximum injection time for MS1 and ddMS2: 100 ms and 45 ms; automatic gain control (AGC) for MS1 and ddMS2: 3e6 and 2e5; isolation window: 1.6 *m*/*z*; normalized collision energies (NCE): 10, 17, 25 or 30, 40, 50). Then, the samples were separated on an amide column (3.5 *μ*M, 2.1 × 100 mm) using water mixed with 25 mM ammonium acetate as mobile phase A and 25 mM ammonium hydroxide mixed with ACN as mobile phase B. The injection volume was 4 *μ*L, and the flow rate was 0.4 mL/min.

### 2.4. Data Processing and Analysis

Compound Discoverer (2.0.0.303) was used to process raw HPLC-MC data, including the extraction of peak statistics, retention time correction, and grouping. An R script was used for signal drift correction for compound quantification. By fitting a local quadratic regression model to correct for signal drift and batch effects, the median peak values were obtained and are shown in the peak table. All missing values, zero values, and negative values were replaced with half of the smallest positive value in the default data set. All metabolites were identified according to the MSI guidelines. We identified metabolites using MzCloud (ddMS2) and ChemSpider (formula or exact mass) databases. MzCloud was compared with ddMS2 (secondary mass spectrometry), while ChemSpider was compared with the molecular formula and mass number obtained by CD software.

An orthogonal partial least-squares discriminant analysis (OPLS-DA) was used to identify differentially expressed metabolites between the GDM and control groups. PLS regression was performed using the PLSR function in R. As a supervised multidimensional statistical analysis method, OPLS-DA was used to identify differences between sample groups and to obtain metabolites with potentially significant differences. A paired *t*-test was used for metabolic signature discovery. Metabolites with variable importance in projection (VIP) values > 1.0 in the OPLS-DA model and *p* < 0.05 by a paired *t*-test were considered significantly different between the GDM and control groups. A false discovery rate (FDR) of <0.1 was used to correct for multiple comparisons. The *q* value in the FDR control was defined as the FDR analog of the *p* value [26]. Metabolites with significant differences were further screened using a *q* value threshold of <0.05. The main parameters determining the quality of the OPLS-DA model are *R*^2^*Y* and *Q*^2^, which represent the interpretation rate and prediction rate of the model, respectively.

### 2.5. KEGG Pathway Analysis

Pathway analyses were conducted using the Kyoto Encyclopedia of Genes and Genomes (KEGG) Pathway Database. KEGG IDs were matched with metabolites in the database and input into MetaboAnalyst (http://www.metaboanalyst.ca/faces/upload/PathUploadView.xhtml) to identify the metabolic network and changes in metabolic pathways related to GDM.

## 3. Results

### 3.1. Demographic and Clinical Characteristics

There were no significant differences in maternal age, gestational age at the time of investigation, gestational age at blood sampling, gravidity, and parity between patients with GDM and controls. However, early pregnancy weight, body mass index (BMI), blood pressure (SBP/DBP), and history of GDM were significantly higher in the GDM group than in the control group (*p* < 0.05). In terms of biochemical markers in early pregnancy, the levels of HGB and LDL were higher in patients with GDM than in controls (*p* < 0.05). Detailed information regarding demographic and clinical characteristics is shown in Table 1.

### 3.2. Quality Control

**Figure 1:**
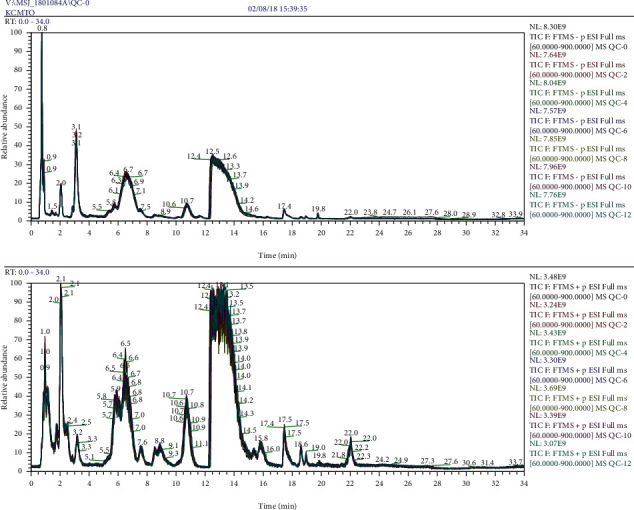
TIC for sequentially selected QC samples.

**Figure 2:**
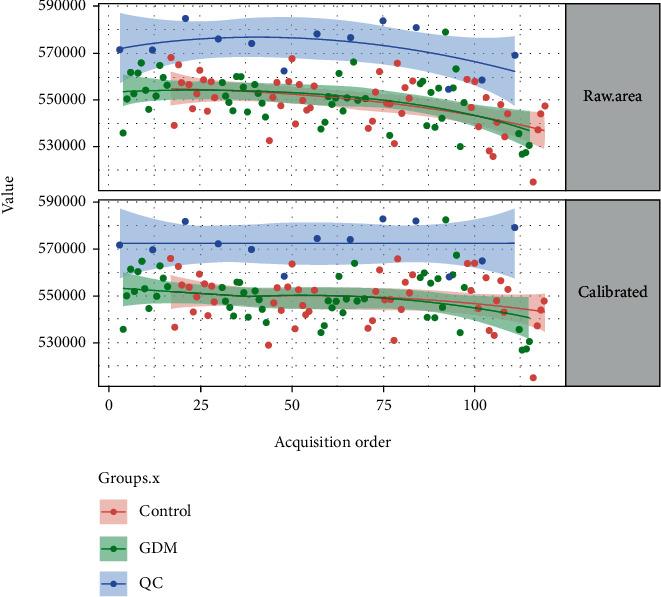
Intensity correction.

QC samples were used to evaluate the repeatability and stability of measurements.  Figure 1 shows a total ion chromatogram for QC samples. The QC samples showed good peak fitting, good data repeatability and instrument stability, and high reliability. The intensities were corrected for signal drift and batch effects by fitting a locally quadratic (LOESS) regression model to the median intensity of pooled QC samples. The alpha parameter (span) controlling smoothing was set to 2 to avoid overfitting. After correction, the median areas of all pooled QC samples were the same. Metabolites with a coefficient of variation in QC samples of >25% were then filtered (6% filtered) owing to their unstable quantifiability (see Figure 2).

### 3.3. Multivariate Data Analysis

**Figure 3:**
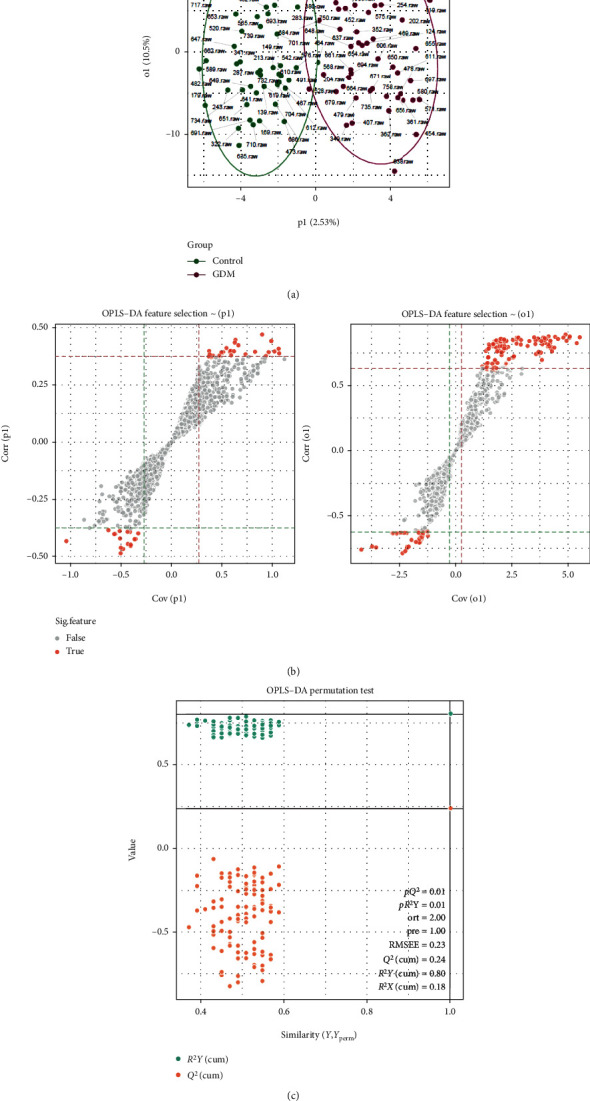
Metabolite profile of the GDM group vs. the control group. (a) Score plot of the OPLS-DA model obtained from GDM and healthy subjects. Separation in *X*(p1) represents between group variation, and separation in *Y*(o1) represents within group variation. (b) The *S*-plot of OPLS-DA. (c) OPLS-DA permutation test at FDR 0.1 level.

In this study, 102 serum samples were evaluated by HPLC-MS, and 2035 characteristic peaks were detected. In order to better distinguish the differences between sample groups and obtain the metabolite information with potential significant differences, OPLS-DA was used to detect metabolic differences between the GDM and control groups. The OPLS-DA score plot (Figure 3(a)) showed that the intragroup difference threshold for metabolites in the GDM and control groups was 10.5% and was mainly explained by variation among individuals. The significance threshold for metabolite differences between the GDM and control groups was 2.53%.  Figure 3(b) showed a scatter plot of model covariance and model correlation combinations from the OPLS-DA model (using Corr > 0.25 and Cov > 0.5). The model quality parameters were *R*^2^*X* = 0.18, *R*^2^*Y* = 0.80, and *Q*^2^ = 0.24. The OPLS-DA model showed a good degree of differentiation (*R*^2^*Y* = 0.80) and was relatively stable and reliable. However, the prediction rate was less than 0.50 (*Q*^2^ = 0.24), indicating that the prediction error of the model was high. A permutation test showed that the *R*^2^*Y* (*pR*^2^*Y* = 0.01) and *Q*^2^ (*pQ*^2^ = 0.01) values for groups obtained by random sampling were less than those of the original model, indicating that the model has high accuracy and reliability, with significant differences between groups (Figure 3(c)).

### 3.4. Identification of Differential Metabolites

In total, 44 metabolites with significant differences between groups were identified using VIP > 1.0 and *p* < 0.05 as thresholds (Table 2). These substances were related to the metabolism of lipids, amino acids, sugars, vitamins, nucleotides, and purines, to various degrees (fold change values, 0.78–1.42). After correction for multiple hypothesis testing (FDR < 0.1), 26 highly significant differential metabolites (*q* < 0.05) were obtained. Of the 26 significant metabolites, 4-oxoproline, dihydrothymine, 1,5-anhydro-D-glucitol, leu-leu, met-val, hexadecanedioic acid, and calcitriol were more abundant in the control group than in the GDM group (*R*‐fold < 1). In contrast, L-glutamic acid, L-pyroglutamic acid, L-cysteinesulfinic acid, xanthine, 2-methylhippuric acid, pantothenic acid, and incadronic acid were more abundant in the GDM group than in the control group (*R*‐fold > 1). See Table 2 for details.

### 3.5. Metabolic Pathway Analysis

**Figure 4:**
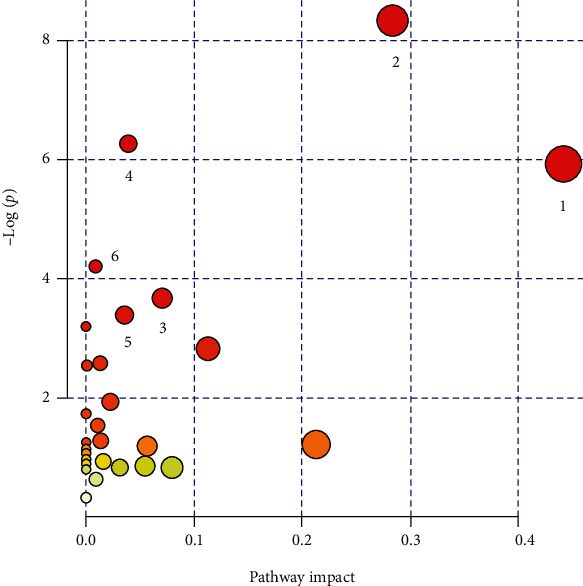
Analysis of metabolic pathways associated with GDM with MetaboAnalyst ((1) alanine, aspartate, and glutamate metabolism; (2) D-glutamine and D-glutamate metabolism; (3) caffeine metabolism; (4) arginine and proline metabolism; (5) pyrimidine metabolism; (6) histidine metabolism).

A total of 15 related metabolic pathways were obtained by a KEGG enrichment analysis (Table 3). Among these metabolic pathways, amino acid (including other amino acids) metabolism was the main pathway, followed by carbohydrate metabolism, lipid metabolism, energy metabolism, nucleotide metabolism, and cofactor and vitamin metabolism. In addition, D-glutamine and D-glutamate metabolism and alanine, aspartate, and glutamate metabolism had the highest enrichment coefficients (Figure 4). The differential metabolites corresponding with the potential metabolic pathways were mainly xanthine, L-glutamic acid, 4-oxoproline, 4-acetamidobutanoic acid, dihydrothymine, pyroglutamic acid, and phenylacetic acid. These differential metabolites and their corresponding metabolic pathways in early pregnancy may be related to the subsequent development of GDM.

## 4. Discussion

In this prospective study, we performed a nested case-control study of GDM using an HPLC-MS untargeted metabolomics approach. By multivariate statistical analysis, we identified 44 significant differential metabolites associated with the risk of GDM. Of these, 26 metabolites differing significantly between the GDM and control groups were obtained after FDR analysis. Our results suggest that in early pregnancy, the serum levels of pantothenic acid, phenylacetic acid, and xanthine are significantly elevated and that of 4-oxoproline is significantly decreased, indicating that these molecules are potential predictors of GDM.

Various differentially expressed metabolites, such as pantothenic acid, L-pyroglutamic acid, L-glutamic acid, phenylacetic acid, and xanthine, were significantly elevated in the GDM group compared with controls. GDM and T2DM have pathophysiological similarities and are expected to share similar metabolic profiles [27]. L-Pyroglutamic acid, L-glutamic acid, phenylacetic acid, and pantothenic acid are each associated with GDM or T2DM. Serum levels of pantothenic acid in patients with T2DM and high BMI are higher than those in normal controls [28]; however, increased serum levels of pantothenic acid have not been reported in GDM. Pantothenic acid, a component of coenzyme A, is involved in the metabolism of carbohydrates, fatty acids, proteins, and gluconeogenesis as a cofactor for a variety of enzyme-catalyzed reactions. A pantothenic acid deficiency can lead to metabolic alterations, including a loss of the eosinopenic response to adrenocorticotropin (ACTH) and increased sensitivity to insulin [29]. Li et al. examined early changes in the development of insulin resistance via liver and plasma metabolome analyses and found that increased pantothenate may be associated with insulin resistance [30]. Thus, the observed increase in pantothenic acid may be associated with changes in insulin sensitivity and insulin resistance, thereby increasing the risk of GDM; however, the underlying mechanism needs to be further studied. Kim et al. reported that L-pyroglutamate, an insulin-like substance that inhibits epinephrine-induced fat breakdown and promotes fat synthesis from glucose, is significantly increased in the peripheral blood of patients with T2DM with impaired fasting blood glucose [31]. However, a GC-MS analysis of a Western population has shown that L-pyroglutamic acid levels are decreased during early pregnancy in patients with GDM [32], inconsistent with our results. This difference may be explained by differences in diagnostic criteria for GDM, differences in metabolome profiling platforms, differences in study populations, or other factors. Therefore, further studies are needed to provide a theoretical basis for the link between L-pyroglutamic acid and GDM in early pregnancy. L-Glutamic acid enhances islet function and increases insulin secretion. Our results showed that serum L-glutamic acid levels are significantly increased in early pregnancy in patients with GDM, consistent with previous results obtained by Zhao et al. [24], suggesting that the decrease in insulin sensitivity occurs earlier in pregnant women with GDM than in healthy pregnant women, which in turn promotes increased L-glutamic acid metabolism and increased insulin compensatory secretion. We obtained the evidence that the level of serum xanthine is significantly increased in the GDM group during early pregnancy. Xanthine is an intermediate product of the purine metabolic process and can be further metabolized to uric acid by xanthine oxidase. The increased xanthine levels in the serum of patients with GDM reflect impaired xanthine oxidase activity. Xanthine oxidase is an important indicator of oxidative stress [33], which can increase inflammatory cytokines, leading to placental damage, insulin resistance, and the occurrence of GDM [34]. Accordingly, we speculate that patients with GDM have an impaired antioxidant capacity before abnormal glucose metabolism. A previous study confirmed that patients with T2DM have elevated phenylacetic acid levels in the peripheral blood [31], suggesting that increased phenylalanine acid levels are related to an increased risk for the development of T2DM. Consistent with this, we observed elevated serum phenylacetic acid levels in pregnant women with GDM in early pregnancy. This may be due to a compensatory increase in serum phenylacetic acid levels in patients with GDM during early pregnancy. Cellular and in vivo experiments have shown that phenylacetic acid can significantly inhibit gluconeogenesis and increase blood glucose by inhibiting pyruvate carboxylase (promoting islet cell activity) [35]. However, the underlying mechanisms need to be further studied.

We also identified various differentially expressed metabolites, such as 1,5-anhydro-D-glucitol (1,5 AG), calcitriol, and 4-oxoproline, showing significant decreases in the GDM group during early pregnancy compared to the controls, and these may be associated with the subsequent onset of GDM. For example, 1,5 AG is a major polyol in humans and is structurally similar to glucose; it is a sensitive and reliable marker of short-term glucose control [36]. Serum levels of 1,5 AG are lower in pregnant women with GDM than in women without GDM [36, 37], consistent with our findings, suggesting that 1,5 AG is a potential marker for the early identification and management of GDM. In addition, 1,5 AG might suppress elevated blood glucose by inhibiting sucrase, lactolytic enzymes, and intestinal glucose absorption [38]. Thus, the decrease in serum 1,5 AG in early pregnancy may reflect a reduction in the inhibition of 1,5 AG via increased blood glucose. Calcitriol is one of the most important active metabolites of vitamin D, which may directly or indirectly regulate *β*-cell function and secretion and enhance insulin sensitivity [39]. Previous studies have shown that a maternal vitamin D deficiency in early pregnancy is associated with an elevated risk of GDM [40, 41]. Consistent with the results of these studies, we found decreased calcitriol levels in patients with GDM in early pregnancy, which may be related to insulin resistance and impaired insulin secretion during pregnancy, subsequently increasing the risk of GDM. To the best of our knowledge, only one study has evaluated 4-oxoproline in T2DM, showing that 4-oxoproline can predict the treatment response of T2DM to metformin and that low 4-oxoproline is associated with a significant decrease in glycated hemoglobin (HbA1c) [42]. We speculate that the decreased 4-oxoproline level may be related to insulin resistance during pregnancy. However, further studies are needed to determine whether the low level of 4-oxoproline in the GDM group is the result of glucose regulation in the compensatory period or other factors. The metabolites described above represent only a portion of the differential metabolites identified in this study, and relationships between other metabolites and the risk of GDM need to be further explored, especially the significant metabolites obtained by secondary screening, such as dihydrothymine, L-cysteinesulfinic acid, met-val, and hexadecanedioic acid.

Furthermore, 15 related metabolic pathways were obtained by a KEGG enrichment analysis, including amino acid metabolism, carbohydrate metabolism, lipid metabolism, energy metabolism, nucleotide metabolism, and cofactor and vitamin metabolism. Recent evidence suggests that amino acid metabolism is closely related to insulin resistance, T2DM, and GDM [43, 44]. In this study, pathways associated with significantly altered metabolites with large impact coefficients were mainly involved in alanine, aspartate, and glutamate metabolism and D-glutamine and D-glutamate metabolism. Vangipurapu et al. performed a large prospective population-based cohort and found that alanine, aspartate, and glutamate are significantly associated with decreases in insulin secretion and elevations of fasting or 2 h glucose levels [45]. Changes in serum metabolites disrupt alanine, aspartate, and glutamate metabolism, affecting insulin tolerance and insulin secretion, which may be related to the subsequent occurrence of GDM. Glutamine is an effective glucose progenitor that stimulates insulin secretion [46]. Andersen et al. reported that glutamate uptake and glutamine metabolism are disrupted in the hippocampus of a T2DM db/db mouse model, potentially affecting the homeostasis of the glutamate/glutamine cycle [47]. Thus, changes in serum metabolites in the early gestational period of GDM may disrupt glutamic acid/glutamine metabolism, affecting the energy balance. Other related metabolic pathways, such as those for lipids, carbohydrates, cofactors, and vitamins, provide insight into GDM-related metabolic changes in early pregnancy and should be evaluated in future studies.

Our study explored differences in the serum metabolic profile in early pregnancy between patients with GDM and healthy controls by untargeted HPLC-MS-based metabolomics techniques in a Chinese population; our approach is beneficial for the identification of specific biomarkers of GDM with predictive and/or diagnostic value in early pregnancy. The analysis of metabolites and metabolic pathways can provide a theoretical basis for future research on the pathogenesis of GDM. However, our study had some limitations. First, the subjects were recruited from a single hospital, limiting the generalizability of the findings. Second, functional studies of some differential metabolites discovered in the study are lacking, and further analyses of the effects of these metabolites are needed. Third, due to the limitations of funds and detection conditions, we only used HPLC-MS instead of UPLC-MS for metabolomic detection and analysis.

## 5. Conclusion

In this study, we identified 44 significantly differentially expressed metabolites associated with the risk of GDM. The levels of L-pyroglutamic acid, L-glutamic acid, xanthine, phenylacetic acid, L-cysteinesulfinic acid, and other metabolites were higher and the levels of 1,5-anhydro-D-glucitol, calcitriol, 4-oxoproline, dihydrothymine, and other metabolites were lower in the GDM group than in the control group, indicating that these molecules are candidate predictors of GDM. Most of the metabolic pathways obtained by a KEGG enrichment analysis are related to amino acid metabolism, suggesting that this process is important for the development of GDM. Of course, further validation of these differentially expressed metabolites in a larger sample population should be considered in the future so as to better elucidate the pathogenesis of GDM.

## Figures and Tables

**Table 1:** Baseline characteristics and clinical information in the case and control groups.

Variables	Case (*n* = 51)	Control (*n* = 51)	*p* value
Gestational age of investigation	12.6 (±0.87)	12.7 (±0.61)	0.503
Gestational age of blood collection	12.7 (±0.99)	12.8 (±0.77)	0.625
Maternal age (years)	32.4 (±4.61)	31.7 (±4.33)	0.494
Early pregnancy weight (kg)	57.7 (±9.27)	51.1 (±7.24)	<0.001
Early pregnancy BMI (kg/m^2^)	23.4 (±3.23)	20.4 (±2.49)	<0.001
Early pregnancy SBP (mmHg)	120.0 (±10.38)	113.7 (±10.07)	<0.001
Early pregnancy DBP (mmHg)	77.0 (±8.55)	72.2 (±7.90)	<0.001
Gravidity	2.5 (±1.22)	2.3 (±1.46)	0.608
Parity	0.6 (±0.61)	0.6 (±0.50)	1.000
History of GDM			0.010
No	43 (84.3%)	51 (100.0%)	
Yes	8 (15.7%)	0 (0.0%)	
History of gestational hypertension			1.000
No	50 (98.0%)	50 (98.0%)	
Yes	1 (2.0%)	1 (2.0%)	
Smoking			0.475
No	51 (100%)	49 (96.1%)	
Yes	0 (0%)	2 (3.9%)	
Drinking			0.475
No	51 (100.0%)	49 (96.1%)	
Yes	0 (0%)	2 (3.9%)	
HGB (g/L)	127.9 (±8.55)	122.9 (±8.78)	0.004
TG (mmol/L)	1.8 (±0.68)	1.5 (±0.72)	0.067
TC (mmol/L)	4.6 (±0.70)	4.4 (±0.70)	0.262
HDL (mmol/L)	1.8 (±0.40)	1.9 (±0.37)	0.079
LDL (mmol/L)	2.6 (±0.60)	2.3 (±0.62)	0.034

^†^Data are *n* (%), means ± SD. ^‡^BMI: body mass index; SBP: systolic blood pressure; DBP: diastolic blood pressure; HGB: hemoglobin; TG: triglyceride; TC: total cholesterol; HDL: high-density lipoprotein cholesterol; LDL: low-density lipoprotein cholesterol.

**Table 2:** The 44 differential metabolites associated with the risk of gestational diabetes.

Metabolite	(*m*/*z*)	RT (min)	VIP	*p* value	*R*-fold
DL-3-Aminoisobutyric acid	103.06	18.998	2.46	<0.001	1.24^∗^
N-Acetylglycine	117.04	4.096	2.53	0.00499	1.38
2-Methyl-3-hydroxybutyric acid	118.06	2.881	1.59	0.00292	0.82^∗^
Dihydrothymine	128.06	16.652	2.06	<0.001	0.80^∗^
4-Oxoproline	129.04	17.506	1.50	0.00231	0.88^∗^
L-Pyroglutamic acid	129.04	19.01	2.02	0.00236	1.18^∗^
Phenylacetic acid	136.05	0.673	2.69	0.01636	1.23
Trimethadione	143.06	3.674	2.46	0.00181	1.23^∗^
4-Acetamidobutanoic acid	145.07	2.844	1.84	0.01210	1.29
(2E)-3-(Carbamimidoylsulfanyl)acrylic acid	146.02	19.827	2.83	0.00221	1.39^∗^
L-Glutamic acid	147.05	18.998	2.35	<0.001	1.23^∗^
L-Cysteinesulfinic acid	153.01	2.579	2.95	<0.001	1.42^∗^
4-Methylquinolin-2-ol	159.07	5.706	2.07	0.00371	1.22
5-Carbamimidamidopentanoic acid	159.10	17.727	3.07	<0.001	1.33^∗^
1,5-Anhydro-D-glucitol	164.07	2.870	1.74	0.00117	0.81^∗^
N-Acetylornithine	174.10	18.644	1.83	0.01884	1.28
Aceglutamide	188.08	18.819	1.51	0.00298	1.20^∗^
Homo-L-arginine	188.13	22.002	1.60	0.00480	1.18
2-Methylhippuric acid	193.07	1.540	2.58	0.00284	1.37^∗^
4-(Nitrosoamino)-1-(3-pyridinyl)-1-butanol	195.10	5.333	2.52	0.01138	1.39
Dinotefuran	202.11	17.917	2.26	0.01146	1.33
Hept-2-ulose	210.07	5.785	1.94	0.00714	1.21
Pantothenic acid	219.11	4.998	2.07	<0.001	1.24^∗^
Eugenitin	220.07	0.631	3.17	0.00796	1.29
Leu-Val	230.16	2.800	2.52	0.00312	0.79
2-Methoxy1,3-thiazino6,5-bindol-4(9H)-one	232.03	19.006	2.30	<0.001	1.22^∗^
Phenobarbital	232.08	1.831	2.08	0.02063	1.31
UNII: 734CNR85EV	234.09	0.667	3.14	0.00960	1.28
4-Phenylbutanoyl hydrogen sulfate	244.04	14.168	1.58	0.00560	1.16
Leu-Leu	244.18	1.753	2.50	<0.001	0.78^∗^
Met-val	248.12	2.852	3.86	<0.001	0.68^∗^
(2R)-2,3-Dihydroxypropyl beta-D-galactopyranoside	254.10	2.961	1.63	0.00731	0.82
Hexadecanedioic acid	286.21	1.456	1.77	<0.001	0.82^∗^
Incadronic acid	287.07	15.528	3.33	<0.001	1.30^∗^
Ethylestrenol	288.25	2.780	2.04	0.00411	1.21
Pentiapine	299.12	15.404	2.33	0.00502	1.17
(9Z,11E,13S,15Z)-13-Hydroperoxy-9,11,15-octadecatrienoic acid	310.21	1.504	1.98	<0.001	0.80^∗^
(9E)-9-Octadecenedioic acid	312.23	1.076	2.41	0.00191	0.79^∗^
Icomucret	320.24	0.819	1.98	0.01263	1.23
Sulfometuron-methyl ANSI	364.08	14.156	1.45	0.00276	1.17^∗^
Calcitriol	416.33	0.815	1.69	<0.001	0.80^∗^
(1S,3R,5Z,7E)-1,3,25-Trihydroxy-9,10-secocholesta-5,7,10-trien-18-yl acetate	474.33	0.853	1.54	0.00286	0.83^∗^
Mupirocin	500.30	2.803	2.14	0.00286	1.21^∗^
Xanthine	76.02	2.799	1.90	0.00174	1.19^∗^

^∗^A false discovery rate (FDR) of <0.1 was used to correct for multiple comparisons. The *q* value in the FDR control was defined as the FDR analog of the *p* value, and the *q* values < 0.05.

**Table 3:** Results of KEGG enrichment analysis of the metabolic pathways related to GDM.

Metabolic pathway	Matched differential metabolites	*p*	−Log(*p*)	FDR	Impact
D-Glutamine and D-glutamate metabolism	L-Glutamic acid	<0.01	8.321	0.019	0.283
Arginine and proline metabolism	L-Glutamic acid, 4-oxoproline, 4-acetamidobutanoic acid	<0.01	6.259	0.072	0.039
Alanine, aspartate, and glutamate metabolism	L-Glutamic acid	<0.01	5.920	0.072	0.442
Histidine metabolism	L-Glutamic acid	0.015	4.203	0.229	0.009
Caffeine metabolism	Xanthine	0.026	3.667	0.409	0.070
Pyrimidine metabolism	Dihydrothymine	0.034	3.382	0.453	0.036
Aminoacyl-tRNA biosynthesis	L-Glutamic acid	0.059	2.823	0.594	0.113
Glutathione metabolism	L-Glutamic acid L-Pyroglutamic acid	0.075	2.584	0.632	0.013
Nitrogen metabolism	L-Glutamic acid	0.079	2.539	0.632	0.001
Alpha-linolenic acid metabolism	(9Z,11E,13S,15Z)-13-Hydroperoxy-9,11,15-octadecatrienoic acid	0.298	1.211	1	0.213
Purine metabolism	Xanthine	0.305	1.188	1	0.056
Butanoate metabolism	L-Glutamic acid	0.387	0.950	1	0
Galactose metabolism	(2R)-2,3-Dihydroxypropyl beta-D-galactopyranoside	0.394	0.931	1	0.016
Phenylalanine metabolism	Phenylacetic acid	0.423	0.860	1	0.054
Porphyrin and chlorophyll metabolism	L-Glutamic acid	0.724	0.3232	1	0

## Data Availability

The metabolomics analysis of the data used to support the findings of this study are available from the corresponding author upon request.

## Supplementary Materials

Supplementary Materials Supplementary Table 1: the 26 differential metabolites associated with the risk of gestational diabetes.
