# Supplementary material for: Serum Metabolites as an Indicator of Developing Gestational Diabetes Mellitus Later in the Pregnancy: A Prospective Cohort of a Chinese Population
Source: J Diabetes Res. 2021 Feb 5;2021:8885954. doi: 10.1155/2021/8885954 (PMC7884125; doi:10.1155/2021/8885954)
Supplement: Supplementary Materials — Supplementary Table 1: the 26 differential metabolites associated with the risk of gestational diabetes. [file 8885954.f1.docx]

**Supplementary material**

**Serum metabolites as an indicator of developing gestational diabetes mellitus later in the pregnancy: a prospective cohort of a Chinese population**

Mengyuan Tian1,2; Shujuan Ma3; Yiping You4; Sisi Long1,2; Jiayue Zhang1,2; Chuhao Guo1,2; Xiaolei Wang1,2; Hongzhuan Tan1,2*

1. Xiangya School of Public Health, Central South University, Changsha, China

2. Hunan Key Laboratory of Clinical Epidemiology, Changsha, China

3. Reproductive and Genetic Hospital of CITIC-Xiangya, Clinical Research Center For Reproduction and Genetics In Hunan Province, Changsha, China

4. Department of Obstetrics, Hunan Provincial Maternal and Child Health Hospital, Changsha, China.

Mengyuan Tian and Shujuan Ma are joint first authors.

* Corresponding author e-mail: tanhz99@qq.com

Supplementary Table 1. The 26 differential metabolites associated with the risk of gestational diabetes

| Metabolite | [m/z] | RT[min] | VIP | P value | R. fold | q value |
| --- | --- | --- | --- | --- | --- | --- |
| DL-3-Aminoisobutyric acid | 103.06 | 18.998 | 2.46 | 0.00024 | 1.24* | 0.02409 |
| L-Pyroglutamic acid | 129.04 | 19.01 | 2.02 | 0.00236 | 1.18 | 0.04748 |
| trimethadione | 143.06 | 3.674 | 2.46 | 0.00181 | 1.23 | 0.04505 |
| (2E)-3-(Carbamimidoylsulfanyl)acrylic acid | 146.02 | 19.827 | 2.83 | 0.00221 | 1.39 | 0.04748 |
| L-Glutamic acid | 147.05 | 18.998 | 2.35 | 0.00022 | 1.23* | 0.02409 |
| L-Cysteinesulfinic acid | 153.01 | 2.579 | 2.95 | 0.00089 | 1.42* | 0.04081 |
| 5-Carbamimidamidopentanoic acid | 159.10 | 17.727 | 3.07 | 0.00020 | 1.33* | 0.02409 |
| Aceglutamide | 188.08 | 18.819 | 1.51 | 0.00298 | 1.20 | 0.04964 |
| 2-Methylhippuric acid | 193.07 | 1.540 | 2.58 | 0.00284 | 1.37 | 0.04936 |
| pantothenic acid | 219.11 | 4.998 | 2.07 | 0.00103 | 1.24* | 0.04095 |
| 2-Methoxy1,3-thiazino6,5-bindol-4(9H)-one | 232.03 | 19.006 | 2.30 | 0.00067 | 1.22* | 0.03563 |
| Incadronic acid | 287.07 | 15.528 | 3.33 | 0.00020 | 1.30* | 0.02409 |
| sulfometuron-methyl ANSI | 364.08 | 14.156 | 1.45 | 0.00276 | 1.17 | 0.04936 |
| Mupirocin | 500.30 | 2.803 | 2.14 | 0.00286 | 1.21 | 0.04936 |
| Xanthine | 76.02 | 2.799 | 1.90 | 0.00174 | 1.19 | 0.04505 |
| 2-Methyl-3-hydroxybutyric acid | 118.06 | 2.881 | 1.59 | 0.00292 | 0.82 | 0.04964 |
| Dihydrothymine | 128.06 | 16.652 | 2.06 | 0.00019 | 0.80* | 0.02409 |
| 4-Oxoproline | 129.04 | 17.506 | 1.50 | 0.00231 | 0.88 | 0.04748 |
| 1,5-Anhydro-D-glucitol | 164.07 | 2.870 | 1.74 | 0.00117 | 0.81* | 0.04095 |
| Leu-Leu | 244.18 | 1.753 | 2.50 | 0.00022 | 0.78* | 0.02409 |
| met-val | 248.12 | 2.852 | 3.86 | 0.00003 | 0.68* | 0.01606 |
| hexadecandioic acid | 286.21 | 1.456 | 1.77 | 0.00081 | 0.82* | 0.03950 |
| (9Z,11E,13S,15Z)-13-Hydroperoxy-9,11,15-octadecatrienoic acid | 310.21 | 1.504 | 1.98 | 0.00057 | 0.80* | 0.03563 |
| (9E)-9-Octadecenedioic acid | 312.23 | 1.076 | 2.41 | 0.00191 | 0.79 | 0.04505 |
| Calcitriol | 416.33 | 0.815 | 1.69 | 0.00003 | 0.80* | 0.01606 |
| (1S,3R,5Z,7E)-1,3,25-Trihydroxy-9,10-secocholesta-5,7,10-trien-18-yl acetate | 474.33 | 0.853 | 1.54 | 0.00286 | 0.83 | 0.04936 |
